# Membrane Separation Coupled with Electrochemical Advanced Oxidation Processes for Organic Wastewater Treatment: A Short Review

**DOI:** 10.3390/membranes10110337

**Published:** 2020-11-12

**Authors:** Kajia Wei, Tao Cui, Fang Huang, Yonghao Zhang, Weiqing Han

**Affiliations:** 1 Key Laboratory of Jiangsu Province for Chemical Pollution Control and Resources Reuse, School of Environment and Biological Engineering, Nanjing University of Science and Technology, Nanjing 210094, China; kajiaw@njust.edu.cn (K.W.); cuitao1992@163.com (T.C.); 19102021666@njust.edu.cn (F.H.); 2 Nanjing Research Institute of Electronic Engineering, Nanjing 210007, China; 3 School of Environmental Science and Engineering, Yancheng Institute of Technology, Yancheng 224051, China

**Keywords:** membrane separation, electrochemical advanced oxidation processes, coupling technology, organic wastewater treatment

## Abstract

Research on the coupling of membrane separation (MS) and electrochemical advanced oxidation processes (EAOPs) has been a hot area in water pollution control for decades. This coupling aims to greatly improve water quality and focuses on the challenges in practical application to provide a promising solution to water shortage problems. This article provides a summary of the coupling configurations of MS and EAOPs, including two-stage and one-pot processes. The two-stage process is a combination of MS and EAOPs where one process acts as a pretreatment for the other. Membrane fouling is reduced when setting EAOPs before MS, while mass transfer is promoted when placing EAOPs after MS. A one-pot process is a kind of integration of two technologies. The anode or cathode of the EAOPs is fabricated from porous materials to function as a membrane electrode; thus, pollutants are concurrently separated and degraded. The advantages of enhanced mass transfer and the enlarged electroactive area suggest that this process has excellent performance at a low current input, leading to much lower energy consumption. The reported conclusions illustrate that the coupling of MS and EAOPs is highly applicable and may be widely employed in wastewater treatment in the future.

## 1. Introduction

Nowadays, membrane separation (MS) and electrochemical advanced oxidation processes (EAOPs) are important technologies in water treatment due to their high removal efficiency, environmental friendliness, easy automation, and low land occupation [1,2,3,4]. How to solve fouling and overcome mass transfer limitation is the key to achieving their wide engineering application [5,6].

Coupling MS and EAOPs together offers a valuable route to solve these critical problems. Treating wastewater with EAOPs before MS is known to improve the influent quality for MS. Likewise, setting EAOPs after MS is commonly used for the treatment of membrane concentrate to weaken the mass transfer limitation of EAOPs. Notably, coupling MS and EAOPs as an integrated technology can further magnify each of their advantages, acquiring both the favorable mass transfer efficiency of EAOPs and antifouling function of MS, which gives great potential in the field of water pollution control.

The present review summarizes recent developments in coupling technology for MS and EAOPs. This paper discusses the combination of MS and EAOPs as a two-stage process (MS -EAOPs or EAOPs-MS) with a focus on the interaction between them and treatment performance. This paper also elaborates the use of MS and EAOPs in a one-pot process by dividing them into two categories, electrochemical anodic membranes (EAMs) and electrochemical cathodic membranes (ECMs), with a discussion of their development histories, advantages, and energy consumption. Then, some application examples of both two-stage processes and one-pot processes are presented to gain deep insight into their prospects for water treatment in the future.

## 2. Coupling MS and EAOPs as a Combined Process

The use of MS in water reclamation plants (WRPs) has developed rapidly due to its ability to separate components with different sizes or physical/chemical properties; examples include particle filtration (PF), microfiltration (MF), ultrafiltration (UF), nanofiltration (NF), reverse osmosis (RO), forward osmosis (FO), membrane distillation (MD), and membrane bioreactors (MBRs) [7,8]. WRPs are constructed with the objective of reusing wastewater for different purposes, such as recreational, agricultural, or industrial purposes [9]. Thus, MS is often used as a tertiary treatment to produce high-quality water that is suitable for recycling or reclamation after removing organic contaminants, suspended solids, and other substrates by biological treatment [10]. However, MS often suffers from its own “Achilles heel”. During operation, the interception and accumulation of contaminants on the membrane surface or inside the membrane pores will result in membrane rejection and fouling [11], which inevitably deteriorates membrane performance and life [12]. For the treatment of most chemical–industrial wastewaters, setting EAOPs prior to MS has been regarded as a good idea and can considerably reduce membrane fouling due to the removal of organic contaminants in EAOPs.

In addition, MS is essentially a physical process of obstruction and concentration, which means that contaminants are not actually “degraded”. Therefore, it is impossible to achieve complete purification simply by MS when wastewater contains refractory organic pollutants. In contrast, EAOPs can decompose refractory organic compounds into biodegradable by-products or low molecular species [13]. For the past few years, EAOPs have been widely applied in WRPs to degrade organic contaminants such as pesticides [14], textile dyes [15,16], landfill leachate [17], pharmaceuticals [18], and explosive chemicals [19]. Although EAOPs are an effective method of addressing toxic contaminants, drawbacks remain due to their high energy consumption caused by the low efficiency of mass transfer. Notably, concentrate from wastewater treated by MS has a relatively high concentration, which can alleviate mass transfer limitations. Moreover, the electrolyte concentration increases during the MS process, which raises the whole system’s conductivity, leading to a significant reduction in energy consumption [20,21]. In this case, one way to overcome challenges by developing mature technologies for commercial applications could be to combine MS and EAOPs. Herein, MS and EAOPs stand as separate units to form a two-stage process of EAOPs-MS or MS-EAOPs. To date, many researchers have studied and reported many synergistic designs of such coupled processes for industrial wastewater treatment. 

### 2.1. MS Combined with EAOPs

**Figure 1:**
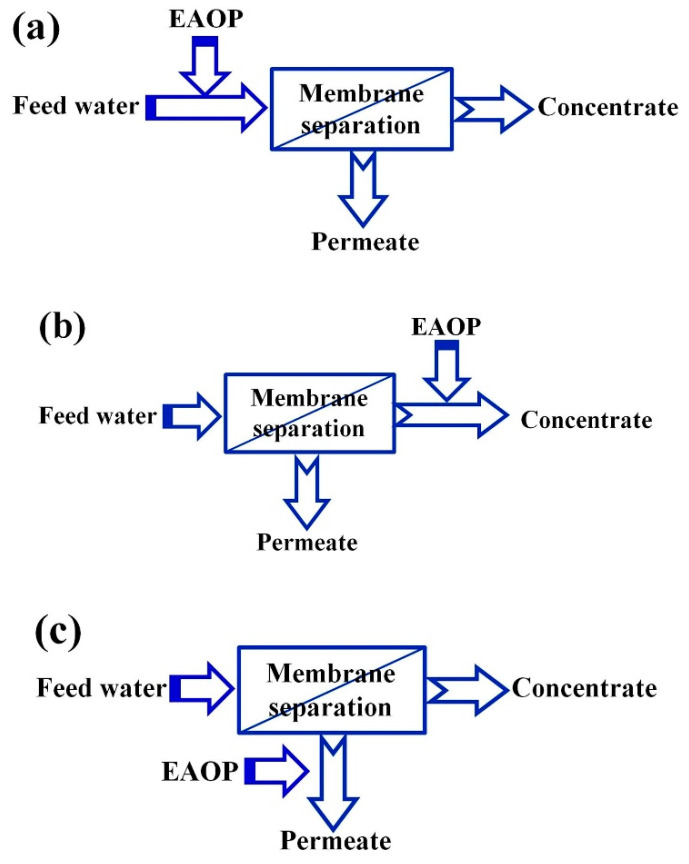
Coupling of membrane processes with electrochemical advanced oxidation processes (EAOPs, the two-stage processes): (**a**) Pre-treatment of feed; (**b**) Post-treatment of concentrate; (**c**) Advance treatment of permeate [3].

Figure 1 presents the combination strategy of MS and EAOPs; either MS or the EAOPs can serve as a pretreatment for the other. When EAOPs are applied before MS, the purpose is to degrade organic contaminants to relieve the treatment load of the membrane. When EAOPs are applied after MS, the aim is to deeply treat the membrane concentrate to meet the discharge standard or treat permeate water to further improve the quality for reuse [1].

#### 2.1.1. MS as Post-Treatment after EAOPs (EAOPs-MS)

The combined process of EAOPs-MS appears promising for wastewater treatment due to its highly efficient removal of organics and salinity. Chen et al. [22] set up a single-cell electrochemical reactor with a PbO_2_ anode and arc-shaped transfer-flow membrane module in alternating sequence to treat wastewater from a textile dye house. Electrochemical oxidation (EO) and MS processes complemented each other, as EO effectively removed the chemical oxygen demand (COD) and chroma and MS nearly completely removed total suspended solids (TSS). Masid et al. [23] investigated the effectiveness of combined treatment processes of coagulation/flocculation (C/F), EO, and membrane processes for tackling the organic load in segregated chemical industry wastewater. Three different combined processes of UF-RO (CP-I), C/F-EO-UF-RO (CP-II), and C/F-EO1-EO2-UF-RO (CP-III) were investigated. The overall COD and total dissolved solids (TDS) removal efficiency was in the order CP-III (93% and 87%) > CP-II (84% and 85%) > CP-I (73% and 82%). These results showed that the EO process considerably reduced the organic load in the effluent, while a large part of TDS removal was attributed to the final RO treatment. Diogo et al. [24] established a combined electrochemical/membrane filtration process for the treatment of wastewater containing phenol and an azo dye (Acid Orange 7, AO7). A boron-doped diamond (BDD) electrode was used as the anode in the electrochemical treatment, followed by RO and NF membranes, which were used in the concentration step. Even though the COD removal in both cases was more than 95%, the combined process only targeted soluble organic pollutants, not suspended particulates.

The above research studied two-stage combination processes. However, with the development of EO and MS hybrid systems, an increasing number of studies have focused on single reactors. Xu et al. [25] reported interesting work on a single reactor where a mesh catalytic electrode was placed on the intercept side of an NF membrane to build a coupled system for dye wastewater treatment. Experiments showed that electroosmosis, electrophoresis, and EO effectively restrained membrane fouling and concentration polarization. Moreover, a high permeate flux was obtained under a relatively low cross flow velocity and pressure. This work provided a novel way to reduce the investment in equipment and the cost of membrane cleaning and replacement while reducing the operating pressure and area of the membrane. Another single reactor was designed by Juang et al. [26], who assembled an EO/MS hybrid system using a Ti/BDD electrode and ceramic membrane to remove Acid Yellow 36 (AY-36) in dye wastewater. Complete COD removal and a more than 90% reduction in turbidity and chroma were achieved. Scaling up laboratory systems to satisfy practical application needs is often associated with an increase in energy consumption, which is an urgent problem to solve. For this purpose, Madsen et al. [27] investigated the reduction in the energy consumption of EO when combining the process with MF, NF, and RO membranes and provided a realistic estimate of the energy consumption during treatment. The pesticide residue 2, 6-dichlorobenzamide (BAM) was used as the target pollutant. The results showed that membranes significantly reduced the energy consumption of the EO process. When using an RO membrane with a recovery flux of 90%, the energy consumption was 95% less (0.96 kWh m^−3^) than that of single EO treatment (18.5 kWh m^−3^). Subsequently, with the breakthroughs of the energy consumption and membrane fouling bottlenecks, the combined process of MS and EO at the laboratory scale and pilot scale has been extensively studied. Mameda et al. [28] designed a new membrane-electrode hybrid reactor (MEO) at the pilot scale as a tertiary treatment for secondary textile wastewater effluent with a particular focus on controlling membrane fouling. The MEO achieved substantial removal of color (50–90%), turbidity (>90%), bacteria (>4 log), COD (13–31%), and 1,4-dioxane (≈25–53%). Furthermore, the pilot-scale test confirmed a substantial delay in membrane fouling (by more than 40 times) at a current density of 150 A m^−3^ compared to the control group. Du et al. [29] reported other interesting work using the PMS-assisted EO/electrolytic coagulation (EC) process as a pretreatment to improve water quality and reduce the fouling behavior of a combined ceramic UF process. Sulfamethazine (SMZ) and organic matter were degraded into small-molecule organics by sulfate radicals (SO_4_^2−^) or hydroxyl radicals (·OH), which led to a significantly lower membrane fouling rate. In particular, this process could achieve stable permeation and retain better filtration performance in the case of high antibiotic and organic matter contents in real surface water treatment.

#### 2.1.2. MS as Pretreatment Followed by EAOPs (MS-EAOPs)

MS can also be used as a pretreatment process before EAOPs. The system can be divided into two configurations depending on whether the EAOPs are applied to treat the membrane permeate or concentrate. This technique has great significance for energy savings and environmental protection, as the treated permeate can be reused for production to reduce the consumption of raw water and the treated concentrate can be directly discharged to the environment.

For permeate treatment, a common example is to use UF or loose NF membranes to reduce water turbidity before the water enters EAOPs. Acosta-Santoyo et al. [30] reported a novel combined process with UF as a preconcentration stage followed by electrochemical degradation of oxyfluorfen with BDD anodes. The efficiency of oxyfluorfen degradation by EO increased with current density, while the degradation of total organic carbon (TOC) followed an opposite trend. According to their results, it would be worthwhile to use UF as a concentration stage for commercial formulations of nonpolar organochlorines due to the high rejection and flux. However, a concentration degree of 2.3 by UF is not enough to decrease the power consumption needed to remove oxyfluorfen by EO. This result could be attributed to the lower proportion of surfactant after the UF process, which hinders the production of persulfate. Mostafazadeh et al. [31] investigated the treatment and reusability of laundry wastewater using UF as the first step followed by EO. Under optimum conditions, UF could achieve 50%, 95%, 97%, and 75% rejection of COD, TSS, turbidity, and nonylphenol ethoxylate (NPEO_3-17_, respectively). Moreover, UF permeate treated by EO with a current density of 12 A and a treatment time of 45 min using a BDD anode and graphite cathode achieved a COD value lower than of 80 mg L^−1^, suggesting the potential possibility of reuse.

For concentrate treatment, NF and RO are often followed by EAOPs to deeply treat the concentrate prior to disposal. Many studies have proven that this technique exhibits excellent performance with several advantages, such as low energy consumption due to excellent conductivity after NF or RO and the possibility of indirect bulk oxidation through the electrogeneration of strong active chlorine oxidants [32,33]. Van Hege et al. [34,35] published a pioneering study that investigated the reduction of recalcitrant organic constituents as well as the removal of total ammonia nitrogen in RO retentate by electrochemical treatment using RuO_2_ and BDD electrodes. Analysis of the inorganic chlorinated species revealed that the oxidation mechanism was mainly due to the indirect oxidative action of electrogenerated hypochlorite. Chaplin et al. [36] reported that the electrochemical destruction of N-nitrosodimethylamine (NDMA) was achieved in RO concentrate (ROC) containing high concentrations of dissolved organic carbon (DOC) and hydroxyl radical scavengers. The destruction half-lives of 3.1 and 6.5 min indicated that a practical system for removing organic matter from ROC or other wastewaters may be feasible. Soriano et al. [37] investigated the treatment of NF concentrate by EO, and the system effectively achieved the removal and mineralization of perfluorohexanoic acid (PFHxA). The electrochemical degradation rate of PFHxA reached 98%, while the energy consumption (15.2 kWh m^−3^) was minimized by selecting an operation parameter of 50 A m^−2^. Kateb et al. [38] studied the mineralization and biodegradability enhancement of NF concentrate from landfill leachate by EAOPs. Their results showed that the most efficient treatment strategy appeared to be heterogeneous electro-Fenton (EF) at 4.2 mA cm^−2^ combined with anodic oxidation using a Ti_4_O_7_ anode (energy consumption = 0.11 kWh (g DOC)^−1^). However, it should be noted that enhanced nitrification is required because of the release of NH_4_^+^ from the mineralization of refractory organic nitrogen by EAOPs.

### 2.2. MBRs Combined with EO

Among all MS, MBRs are special because of the involvement of biological treatment. MBRs have been proven to be an efficient method of wastewater treatment and reclamation in recent years owing to the benefits of effluent quality, process control, and compatibility with various types of wastewater. However, an inevitable fouling problem limits the application of this technology in full-scale practical operation [39,40]. A considerable body of literature has reported the use of EO as a viable method to alleviate membrane fouling in MBRs by an in situ generation of free chlorine species. Chung et al. [41] introduced perforated Ti/IrO_2_ anodes and Ti/Pt cathodes into a conventional MBR system with a microfiltration module (EO-MBR). The membrane fouling characteristics of the EO-MBR were monitored for approximately 2 months for comparison with the control groups. The results showed that the in situ generation of free chlorine by EO could alleviate physically irremovable fouling and reduce the required frequency of membrane maintenance. Gurung et al. [42] investigated the EO of synthetic solutions containing carbamazepine (CBZ) and real MBR effluent using newly developed Ti/Ta_2_O_5_-SnO_2_ electrodes. After optimization of the operation parameters, the removal of CBZ (20 mg L^−1^) and TOC was 75.5% and 71.1%, respectively. The EO process based on the Ti/Ta_2_O_5_-SnO_2_ electrode was found to be a reliable approach to remove CBZ when integrated with an MBR. Zhang et al. [43] provided evidence that the total energy consumption of MBR processes for the treatment of municipal wastewater is between 2 and 2.4 kWh m^−3^, while the operating cost of EO-MBRs is only 1% of this value [42]. However, such a combination of EO-MBR processes is still in its infancy stage, and more studies are needed to clarify the mechanism.

The combination of EAOPs and MS in a two-stage process has been proven to be an effective method of organic wastewater treatment. This combined process can not only improve water quality but also reduce energy consumption. However, difficulties in scale up and the lack of a deep understanding of the mechanism still restrict such combined processes to the laboratory scale, and the reduction in energy consumption cannot satisfy current demands for engineering applications. Comparatively, the coupling of MS and EAOPs into an integrated technology (or one-pot process) has attracted more attention than two-stage processes, not only in consideration of practical applications but also the benefits to MS and EAOPs themselves.

## 3. Coupling MS and Anodic EAOPs as an Integrated Technology

### 3.1. Development of EAM Technology

**Figure 2:**
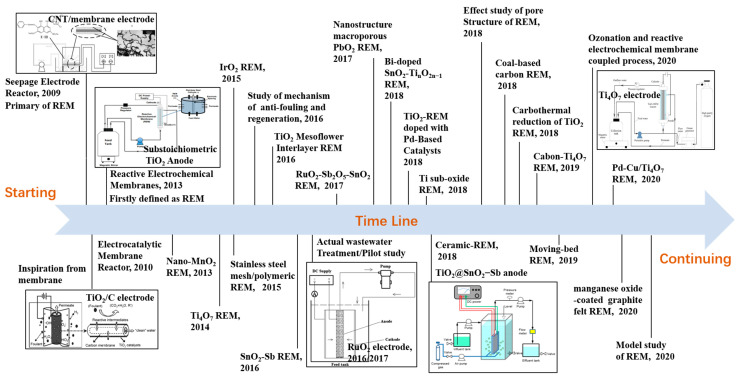
Schematic figure of brief development history of a reactive electrochemical membrane (REM) during 2009–2020 [2,5,6,44,45,46,47,48,49,50,51,52,53,54,55,56,57,58,59,60,61,62,63,64,65,66,67,68,69,70,71,72,73].

The most important EAM technology is the reactive electrochemical membrane (REM), which has appeared in recent decades. This technology couples MS and EO together to solve the critical problems of both processes, such as fouling for the former and mass transfer limitation for the latter [2,3]. A brief history of the development of REM is presented in Figure 2. As we can see, the first instance of REM appeared in 2009 when Yang et al. assembled a reactor by combining a seepage electrode with a porous membrane to improve the mass transfer coefficient [44]. Later, Li et al. reported an electrocatalytic membrane reactor with a self-cleaning function for treating oil wastewater [21]. Chaplin et al. [45] designed an electrochemical reactor equipped with a porous substoichiometric TiO_2_ anode and first defined the system as an REM. Since then, both Li’s and Chaplin’s group developed several REMs and applied them not only in wastewater treatment but also in chemical synthesis [46,47,48,49,50,51,52,53,54,55,56]. Our group has also investigated various REMs in the form of titanium-based metallic oxide electrodes, such as RuO_2_, SnO_2_-Sb, PbO_2_, and RuO_2_-Sb_2_O_5_-SnO_2_, and all electrodes have shown excellent performance for contaminant removal in both simulated and actual wastewaters [14,57,58,59,60,61,62,63]. To date, many kinds of REMs with new catalytic layers have been developed for different purposes (see Figure 2) [2,5,6,44,45,46,47,48,49,50,51,52,53,54,55,56,57,58,59,60,61,62,63,64,65,66,67,68,69,70,71,72,73]. 

Recently, a novel form of REM was designed by Zheng; the reactor is composed of a ceramic membrane with TiO_2_@SnO_2_-Sb Ti mesh to efficiently remove low-molecular-weight anthropogenic contaminants (e.g., *p*-chloroaniline) [69,70]. The introduction of ceramic materials may give a new route for the development of REMs. In addition to those mentioned above, some theoretical studies concerning the antifouling and regeneration mechanism or the effect of pore structure and modeling studies on fouling behavior have been performed to promote the understanding of REMs [70,71,72,73]. In conclusion, REMs have shown excellent performance in the field of water environment control and great potential in other industrial applications due to their unique advantages. For practical applications, their long-term and continuous operation performance deserves to be studied.

### 3.2. Advantages and Mechanisms of REM

#### 3.2.1. Enhancement of Mass Transfer and Electroactive Surface Area

From a mechanistic perspective, EO involves two processes: direct oxidation (direct electron transfer to the anode) and indirect oxidation (indirect or mediated oxidation by ·OH formed from water discharge at the anode). Both direct oxidation and indirect oxidation occur near the anode, because the former requires collision between the pollutant and electrode, and the ·OH of the latter only exists in a narrow zone adjacent to the electrode surface (<1.0 μm) due to its high reactivity and very short life (10^−9^ s) [74,75,76]. However, the mass transfer efficiency of pollutants is mainly dominated by the diffusion performance under a concentration gradient, which is known to be insufficient in traditional EO systems. The conventional way to solve this critical problem is to add flow obstacles to the interelectrode gap to promote turbulence, as proposed by F.C. Walsh [77]. However, this approach still has some inevitable disadvantages, such as the bypassing of reactants to the electrode at a high flow rate and the shielding of the electrode active surface [78]. Fortunately, studies have found that REMs can achieve a significant increase in mass transfer without any loss of active surface area of the electrodes. Two- to ten-fold increases in the mass transfer rate constant (*k_m_*) have been found for different REMs (see Table 1). This enhancement was further theoretically verified by modeling the mass transfer procedure via computational fluid dynamics (CFD) methods [61,79,80]. Moreover, REMs were operated in “flow-through” mode, where all of the high specific surface area of the electrode is activated for electrochemical reactions, and the tiny pores on the REMs can break through radial diffusion limitations [81]. Furthermore, the high flow-through rate derived from the suction of the water pump not only gives fast mass transfer but also produces a more homogeneous velocity distribution and higher turbulent mixing around the electrode, resulting in full contact between the target pollutants and the electrode [82]. Finally, yet importantly, membranes in REM reactors can be simultaneously self-cleaned along with the degradation of pollutants at the electrode surface [81].

In addition to their high mass transfer, another advantage of REMs is their large surface area. Both the highly rough surface and the inner space of the membrane electrode can be utilized in the EO process. Usually, the inner space of traditional plate electrodes consists of cracks in the catalytic layer, which were identified as the “inner surface” by Montilla [83]. These areas are difficult for proton-donating species to access and therefore are useless for EO. However, for porous membrane electrodes, the “inner surface” no longer consists of cracks but instead of membrane pores, which come from the irregular accumulation of titanium particles [57]. Studies have shown that these inner surfaces can also participate in EO, indicating that membrane electrodes have a much larger electrochemical active area than plate electrodes with the same geometrical area [60,83,84]. Electrochemical impedance spectroscopy (EIS) and cyclic voltammetry (CV) are common methods used to measure the electroactive surface area. These methods evaluate the double-layer capacitance or integral area of curves because the electroactive surface area can be estimated by assuming values for the specific capacitance for the former and voltammetric charge for the latter [45,85]. Comparing REMs and traditional EO systems, the outcomes of related studies are breathtaking: the smallest increase in electroactive surface area for REMs was 2-fold, and the largest increase was 619-fold (see Table 1). However, compared to the total surface area results observed by Hg porosimetry or N_2_ adsorption/desorption isotherms, the electroactive surface area calculated by EIS and CV provides an upper bound estimate. These tests indicated that only a fraction of the total surface area of the REMs was electroactive (1.5–11.4%). Trellu et al. considered there to be two probable explanations for this conclusion. The first explanation is low conductivity, which would generate a significant potential drop in the electrode phase, meaning that only a fraction of the surface area can be measured. The second is size exclusion or a lack of interconnectivity, which would cause a large portion of the surface area to be inaccessible to the electrolyte; thus, the electrolyte cannot enter all of the pores [2]. Despite the above discoveries, hardly any cracks form on the catalytic layer of REMs, which is mainly attributed to the compact and uniform distribution of crystal particles on the membrane electrode surface, indicating better electrochemical properties [57,83,84].

Due to their enhanced mass transfer and enlarged electroactive area, REMs are able to effectively degrade pollutants at much lower current density (≈5 mA cm^−2^, and even as low as 0.05 mA cm^−2^) [45,57,58]. Moreover, the higher electrocatalytic activity of REMs assists the fast decomposition of pollutants to greatly relieve fouling, which not only maintains flux but also expands the membrane service life. In addition, these advantages do not change the degradation pathways of pollutant by the involved process (e.g., direct oxidation, indirect oxidation by generated reactive oxygen species (ROS)), but the enhanced mass transfer accelerates these processes as the pollutants transfer to the electrode surface faster; meanwhile, an enlarged electroactive area provides more sites to generated ROS that increase the amount during the degradation. These improvements can perfectly explain the excellent performance of REM at low current density, as was mentioned at the beginning.

#### 3.2.2. Mechanism of Antifouling and Membrane Regeneration

**Figure 3:**
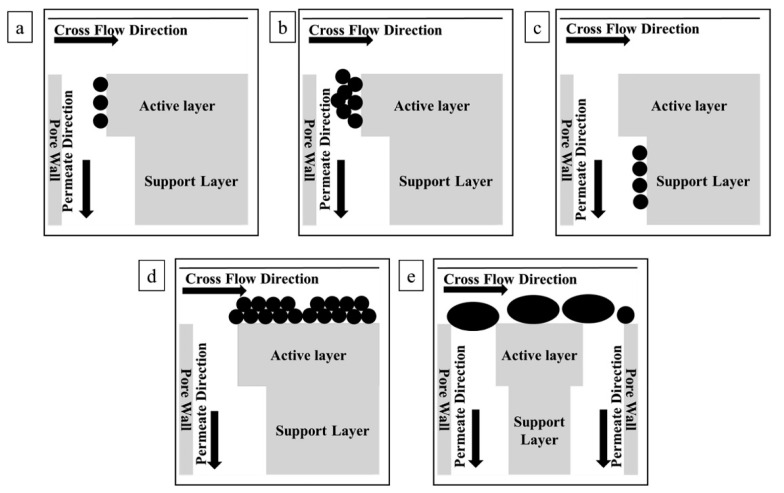
Schematic diagrams of various fouling mechanisms: (**a**) monolayer adsorption; (**b**) pore constriction at the active layer; (**c**) monolayer adsorption at the support layer; (**d**) outer surface fouling; (**e**) intermediate pore blockage [71].

Fouling is the most critical problem that hinders the industrial utilization of MS; fouling increases the transmembrane pressure, reduces membrane life, and causes serious operational challenges. Chaplin et al. proposed a noninvasive and nondestructive method to study membrane fouling on a substoichiometric TiO_2_ REM via EIS [71,72]. As EIS is sensitive to interfacial processes and surface geometry, it is considered to be an effective tool to evaluate fouling and regeneration processes in REMs. By superimposing a small-amplitude alternating potential onto a constant applied potential between the working electrode (e.g., REM) and reference electrode, an EIS signal can be obtained and then calculated with appropriate mathematical models such as the transmission line model (TLM) to spatially characterize the membrane fouling status. According to Chaplin et al.’s studies, fouling appears on the active layer, support layer, and outer membrane surface. This fouling process can be explained by several fouling models, including monolayer adsorption, pore constriction, and intermediate pore blockage (see Figure 3). Therefore, elimination of these foulants will clearly provide antifouling ability, which is the function of EO in REMs. For this case, Yang et al. summarized two possible ways that REMs inhibit fouling [89]. First, EO decomposes foulants into CO_2_ and H_2_O or small biodegradable products. Second, transmembrane microflows from the driving force can hinder foulant adsorption and deposition through the effect of hydrodynamic shear force on the pore structure, which greatly reduces concentration polarization [90]. Fu’s study more specifically explained antifouling behaviors [91]:

1. Repulsion between the foulant and membrane occurs in the presence of an electric field;
2. The diffusion of reactive oxygen species (ROS) and active chlorine (AC) generated by the EO process to the vicinity of the membrane surface helps in membrane cleaning by reacting with foulants;
3. ROS and AC produced in the membrane lumen can achieve in situ membrane cleaning;
4. Foulants are degraded by EO into small molecules that mitigate membrane fouling.

In addition to providing antifouling, the self-cleaning function of REMs is believed by some researchers to help in membrane regeneration [72]. Traditionally, MS regeneration usually utilizes physical and chemical cleaning protocols, such as forward/backwashing and chemical reagent cleaning (e.g., NaOH). However, periodic wash using physical shear forces for the regeneration of membranes cannot completely recover flux. Chemical methods have better flux recovery but high reagent consumption (up to 0.5 mol L^−1^ m^−2^). Moreover, the cost of synthesis, transport, and storage, as well as the risk of environmental damage, pose challenges to the use of chemical reagents, which led to the proposal of a new and chemical-free electrochemical regeneration (CFER) method. REMs could be used as a kind of CFER due to their self-cleaning function through the EO process. Chaplin et al. investigated the membrane fouling of REMs with two foulants, humic acid (HA) and polystyrene microspheres (PM), using different electrochemical regeneration methods, such as forward washing, backwashing, and multiple cycles. The results showed that anodic CFER in forward wash mode could not fully regenerate the HA-fouled REM due to the potential drop with increasing depth in the REM pores. During forward washing, some regions of the active layer and support of the membrane electrode only provide direct electron-transfer treatment of HA, not ·OH oxidation treatment. In contrast, anodic CFER in backwash mode can fully regenerate permeate flux with the assistance of cross-flow shear force. A long-term multicycle test of an anodic CFER in backwash mode was conducted, and the results showed a permeate flux recovery between 76.0 ± 1.1% and 99.0 ± 0.57% under five continuous fouling–regeneration cycles, indicating that anodic CFER in backwash mode is an effective offline cleaning method for HA-fouled membranes. The regeneration of PM-fouled membranes was undertaken in both anodic and cathodic CFER in backwash mode. The results suggest that regeneration is more effective after applying a potential and is equal under both anodic and cathodic conditions. However, the EIS spectrum of the regenerated membrane was not identical to that of the original membrane, indicating that some surface passivation or other changes may have occurred on the REM. In addition, the interaction of electrochemically produced gas bubbles with PM might be the possible reason for the better flux recovery because complete recovery can only be achieved when CFER is conducted under anodic or cathodic conditions. Electrochemical regeneration in backwash mode is able to recover membrane fouling completely, and the advantages of no chemical reagent addition make it a cost effective, time efficient, and environmentally friendly method.

### 3.3. Application of REMs

Due to the advantages summarized above, REMs have been employed in many aspects of wastewater treatment, including organic decomposition, oil removal, bacterial inactivation, and water disinfection. Some researchers have also investigated chemical synthesis and extraction processes with the assistance of REMs. Table 2 summarizes recent REM applications for wastewater treatment. For example, Li’s group undertook many studies of REMs for the removal of organic contaminants and oil from wastewater as well as the synthesis of valuable chemicals [21,46,48,87,89]. They found that EO gives a self-cleaning function to REMs that effectively expands the membrane’s service life, and membrane filtration enhances the oxidation process with respect to mass transfer and the active area, leading to a remarkable pollutant removal rate. Chaplin’s group investigated organic removal with anodic and cathodic REMs, and the results are in accordance with Li’s work [45,49,51,52]. Moreover, Chaplin’s group concluded that REMs can accomplish these targets at a very low current density, which results in much lower energy consumption. Our group has not only studied REMs for the removal of organic contaminants from simulated wastewater but also employed REMs to treat actual wastewater, including a pilot-scale study of a system with a capacity of 10 m^3^ d^−1^ for the treatment of triazole fungicides (TFs) containing discharged water [14,58,60]. Similarly, REMs were found to remove organic contaminants efficiently at a low current density, and both the electric energy consumption (EEC) and operating cost were lower than those of other electrochemical reactors. Lab-scale and pilot-scale studies of REMs have confirmed that this method is highly applicable. Other studies on organic removal by various REMs can also be found in Table 2 [44,53,65,69,92]. As we can see, in addition to common applications, some other interesting studies are included. For instance, Gayen et al. applied REMs in denitrification, and excellent performance was observed in that the concentration of NO_3_^−^ (1 mM) was lower than the Environmental Protection Agency (EPA) regulatory MCL (700 μM) after a short treatment time (≈2 s), and further evidence showed low energy consumption for this process [51]. Hua et al. investigated algal cell destabilization and lipid extraction on a Ti_4_O_7_-based REM, and the results suggested that algal cells exhibited significant disruption and the lipid extraction efficiency increased more than 1.5 times after treatment [93]. Bacterial inactivation and wastewater disinfection by REMs were conducted by Liang, Huang, and Lei. They all observed remarkable performance, with a result of 97.2% for Liang’s study and 100% for the rest [94,95,96]. Moreover, REMs have performed well in the removal of low-concentration and trace pollutants. Chen et al. investigated the removal of low-concentration (ng/L–μg/L) antibiotics with a moving-bed electrochemical MBR in which the anode actually was a REM [66]. The results not only showed an 88.9% removal efficiency of sulfamethoxazole (SMX) but also indicated that the applied electric field could increase the richness/diversity of the microbial community, which was potentially capable of mineralizing SMX. Yang et al. developed a β-PbO_2_ REM to remove norfloxacin and SMX at trace concentration levels in both surface water and the final effluent of wastewater. Only a short residence time of 2.0–5.4 s was required to reach log removal with a low energy cost of 0.005–0.024 kWh m^−3^ [97]. By using a Ti/SnO_2_-Sb REM, Zhou et al. undertook a similar study to remove trace concentrations of the antiretroviral drug stavudine from wastewater and obtained a stavudine degradation efficiency of 90% with an EEC ranging from 0.87 to 2.29 Wh L^−1^. A degradation pathway was also proposed with the toxicity discussion in their study [98].

In summary, by solving the critical problems of MS and EAOPs, REMs possess huge advantages in wastewater treatment and water purification, leading to an intense increase in research in this field. However, some aspects of REMs are still worth deep exploration. Since EO only takes place at the surface of the anode, the utilization of the cathode is often ignored. In fact, there is great potential for REM applications of cathodic oxidation (e.g., EF and electroperoxone (EP)) that should be taken into account.

## 4. Coupling MS and Cathodic EAOPs as an Integrated Technology

### 4.1. Development of ECM Technology

ECMs can be classified into cathodic electrochemical filter membranes (CEFMs) and gas diffusion cathodes (GDCs). Similar to the traditional Fenton/ozonation reaction, EF, photoelectro-Fenton (PEF), and EP also aim to produce ·OH through a reaction between H_2_O_2_ and Fe^2+^ or O_3_, which is a strong oxidizing substance (E_0_ = 2.8 V/SHE) that can nonselectively attack most organics [100]. The diffusion of air, O_2_, or a mixture of O_2_ and O_3_ is the key to the output of H_2_O_2_ in these systems. However, the low mass transfer at the solid–liquid interface of normal plate cathodes hinders the cathodic reaction efficiency. Therefore, cathode membrane materials with sufficient pores, such as carbon felt, graphite, gas diffusion electrodes (GDEs), and active carbon fiber, are widely used [101,102,103]. In particular, due to not only their easy fabrication but also their stable H_2_O_2_ generation and low H_2_O_2_ decomposition, carbon-based cathodes are regarded to be more suitable than metal cathodes such as stainless steel and Pt. Liu et al. [104] prepared a cathode film using polyphenylene sulfide (PPS) ultrafine fiber and conductive carbon black (CB), which has a large porosity, good adsorption, and many active sites. The H_2_O_2_ output was greatly improved, and the removal rate of methylene blue (MB) was twice that of stainless steel mesh. Brillas et al. [105] added UV light to an EF–membrane system, and the use of a membrane cathode enhanced the mass transfer efficiency, which effectively improved the mineralization of PEF. Wang et al. [106] introduced ozone into an EF system, and the results indicated that the mass transfer of O_3_ in water increased by two times and that the generation of ·OH was promoted with the assistance of ECM.

### 4.2. Reaction Mechanism of ECM Technologies

#### 4.2.1. CEFMs

**Figure 4:**
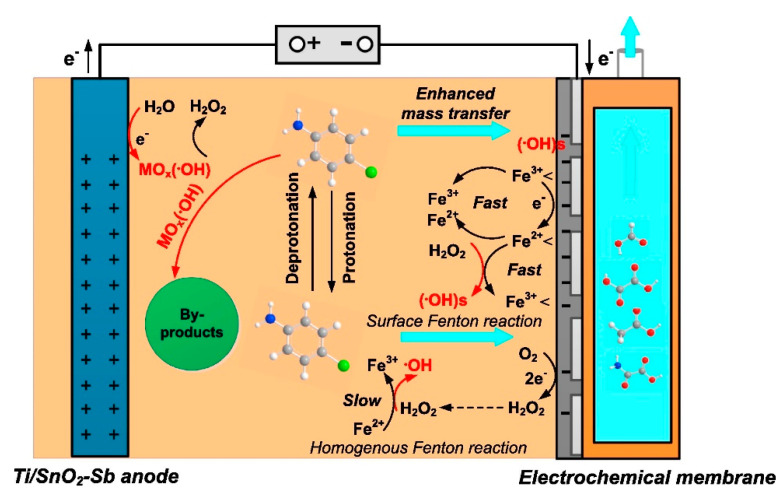
Schematic diagram of cathodic electrochemical filter membrane [107].

Membrane electrodes tend to use materials with high porosity and a large specific surface area (e.g., carbon nanotubes (CNTs)), which can provide relatively high flux at low operation pressures [107] (see Figure 4). Oxygen can be reduced at the cathode to generate hydrogen peroxide in situ and produce highly oxidizing ROS, which could also attack pollutants to decrease TOC and COD. Zheng et al. [108] prepared a porous ceramic microfiltration CEFM. They found that in an EFM system, the mass transfer rate is closely related to membrane flux, and the filtration process increases the mass transfer constant due to better contact of target organics with ROS, which contributes to the more efficient degradation of pollutants. Le et al. [109] prepared a hybrid metal–graphene membrane. Due to the large porosity of the F-rGO membrane (50–55%), a high flux value (approximately 5000 L h^−1^m^−2^) was obtained. A pilot-scale mineralization current efficiency of 165% was achieved by coupling the EF process and filtration; this value was 4.5 times higher than that in the EF bath experiment. As mentioned, filtration membranes always face challenges related to fouling. However, CEFMs, similar to EAMs, can alleviate this problem by indirectly producing ROS, especially ·OH, on the cathode film to degrade fouling pollutants [110].

#### 4.2.2. GDCs

**Figure 5:**
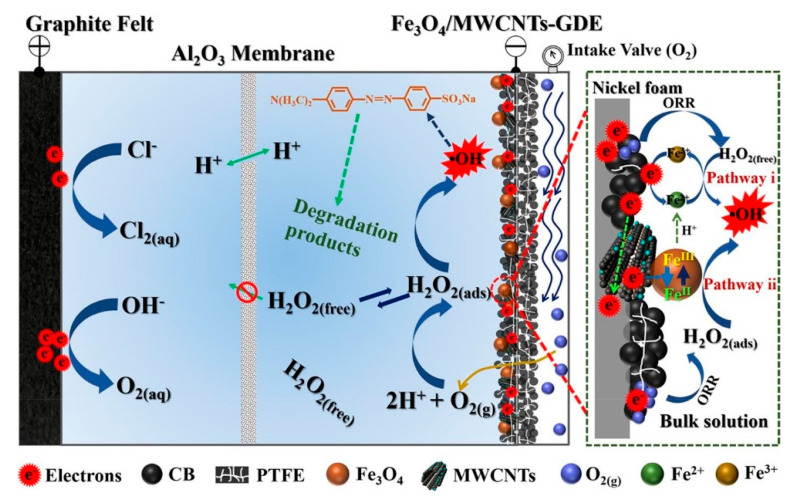
Schematic diagram of gas diffusion membrane cathode [114].

Gas diffusion cathodes (GDCs) could also be considered as cathodic membranes. They can well separate the gas and liquid phases to improve the mass transfer of oxygen (see Figure 5). Li et al. [111] studied the electrocatalytic membrane contact ozonation (ECMCO) process. They found that the application of a GDC enhances the mass transfer of O_3_ and thereby accelerates the production of H_2_O_2_ by the reduction of O_2_ or O_3_ in the electrocatalytic layer. Then, rapidly generated H_2_O_2_ immediately reacts with O_3_ to produce ·OH and thereby improves the whole system’s current efficiency. Usually, cathode membranes are fabricated by a combination of polymers (such as polytetrafluoroethylene (PTFE)) and carbon materials to form a stable structure that also has high conductivity and low resistance. Lu et al. [112] modified a gas diffusion electrode with tert-butyl-anthraquinone (TBAQ). The H_2_O_2_ yield and current efficiency (CE) were stably maintained for six cycles. Moreover, air or oxygen could directly pass through the diffusion layer of the membrane, which improved the oxygen utilization rate. In addition, the thin and porous structure of the membrane is beneficial for injected oxygen or air to seep through the pores and contact oxygen reduction reaction (ORR) active centers, which generates a large amount of H_2_O_2_. Ye et al. [113] prepared Co-based air-diffusion cathodes to enhance the mass transfer function of O_2_, and they also found enhanced electrocatalytic H_2_O_2_ production; moreover, the CE reached 100% at low current.

ECM could make good use of the reduction reactions of the cathodes to generate ROS by reducing O_2_ to H_2_O_2_, thereby achieving the purpose of degrading pollutants. The application of porous cathode membranes improves the mass transfer efficiency of gaseous O_2_, which greatly improves the utilization efficiency of the electrochemical reaction system.

### 4.3. Application of ECMs

Table 3 summarizes recent ECM application for real wastewater treatment. EF membrane cathodes are widely used in the treatment of nonbiodegradable wastewater (see Table 3). Tang et al. [115] used a modified CNT double-layer membrane cathode for the continuous EF oxidation of *p*-nitrophenol. N-doped multi-walled carbon nanotubes (NMCNTs) and CNTs with iron ion compounds with carboxyl groups (CNT-COOFe^2+^) were used to prepare the diffusion layer and catalyst layer of the membrane cathode. After 120 min, the removal rate of *p*-NP reached 96.04% at a cathodic potential of −0.7 V vs. SCE at neutral pH, while a mineralization efficiency of 80.26% was obtained at 180 min. The researchers also tested the stability of the membrane cathode and found that the *p*-NP removal rate was higher than 70%, even after six cycles. Liang et al. [116] used a tubular electrochemically reactive carbon graphite membrane as the cathode to degrade AO7. After 60 min of electrolysis, the degradation efficiency of AO7 dye reached 96% with a low Fe^2+^ concentration (ranging from 0.02 to 0.3 mM). Although EF is effective for the degradation of many contaminants, the energy consumption remains at a high level, which ranges from 87.7 to 275 kWh (kg TOC)^−1^. Gao et al. [117] studied a CNT membrane stack reactor and found that the flow-through EF system had good efficiency (≈45% current efficiency), a low cell voltage (<3.0 V), and low energy consumption (46 kWh (kg TOC)^−1^) at neutral pH. This result indicates that EF membrane technology is cost-effective and therefore has good application prospects. Thiam et al. [118] undertook an urban wastewater treatment experiment and discovered that carbofuran is abated more rapidly in the sequence EO-H_2_O_2_ ≤ EF < PEF with GDC. TOC was only reduced by 4.9% after 360 min by the EO-H_2_O_2_ process, while the reduction obtained with EF reached 36.1%. Comparison of these processes suggested that PEF with GDC has a faster mineralization rate, as it reached 82.4% at 240 min with a final TOC decay of 85.0%. Moreover, the lowest EC values were attained in PEF, decreasing to 540 kWh (kg TOC)^−1^ at 90 min and reaching 1680 kWh (kg TOC)^−1^ at the end of treatment. Some scholars have also used PEF cathode membrane technology to degrade refractory organic wastewater and achieved good application results.

EP processes can mineralize organic pollutants much more effectively than single ozonation. Li et al. [119] used a carbon–PTFE membrane cathode to degrade the anti-inflammatory drug ibuprofen. The results showed that the EP process completely degraded ibuprofen within 7 min, and complete TOC removal was achieved after 2 h with an initial ibuprofen concentration of 20 mg L^−1^. Bakheet et al. [120] decomposed Orange II by an EP process with a carbon–PTFE membrane cathode. After 45 min of EP treatment, complete decolorization and 95.7% TOC removal were obtained, while only 55.6 and 15.3% TOC were removed after 90 min of individual ozonation and electrolysis treatments, respectively. Then, they conducted synthetic dye wastewater treatment experiments and discovered that the EP process can significantly improve the degradation efficiency of Orange II (TOC degradation of ≈90–96% in 30–45 min). Zhang et al. [121] used EP to treat simulated ballast water and found that the inactivation of *E. coli* was an order of magnitude higher than that obtained with ozone oxidation and electrolysis. During the process, the EEC value was 0.33 kWh m^−3^ for BW1 (initial *E. coli* concentration: 106–107 CFU mL^−1^) with an effect of log(c/c_0_) at −5.1, and the EEC decreased to 0.12 kWh m^−3^ when *E. coli* < 250 CFU (100 mL)^−1^ for BW2 (initial *E. coli*: 0.6 × 104 CFU mL^−1^), which was much better than the results obtained using UV (0.91 kWh m^−3^) and UV/Ag-TiO_2_/O_3_ (0.44 kWh m^−3^), if only the cost of ozonation was taken into consideration.

The reaction mechanism of ECMs has not been fully elaborated, and the degradation model of pollutants needs to be established. At present, the preparation method of cathode films is still complicated, which hinders their application to a certain extent. Therefore, how to prepare an economic, efficient, and convenient cathode membrane reactor is important. New technologies or methods also need to consider the economy, stability, and service life of the membrane. In summary, the development of ECM still has a long way to go. Although ECM is not mature at present, it is expected that it will eventually be applied to industry following future research efforts.

## 5. Conclusions and Prospects

The coupling of MS and EAOPs is considered an efficient way to treat industrial wastewater according to the above superiorities. The combined (two-stages) process not only achieves standard discharge of wastewater to remove threats to the ecosystem, but it also could greatly improve the water quality to replace the raw water from the municipal pipe network, which brings favorable economic benefits. The integrated (one-pot) process is regarded as a breakthrough innovation that can separate and degrade organic pollutants simultaneously with high efficiency and low energy consumption, due to its enhanced mass transfer and/or enlarged electro-active area, as well as the much longer service life and easily regeneration procedure. However, there still exist some critical problems that need to be addressed prior to its full-scale application:

1. Development of suitable electrode and membrane materials to acquire better catalytic and physical properties (e.g., OEP, conductivity, corrosion resistance, and impedance);
2. Comprehensive study of coupling mechanism of two technologies to clarify the interaction to explore the optimum operating conditions;
3. Deep understanding of fluid dynamic in the coupling system during the operation, better to do modeling analysis by computer flow dynamic (CFD) study;
4. Necessary engineering optimization of arrangement of facility to simplify the implementation.

These studies are in immediate need to overcome the present problems of the coupling of MS and EAOPs. It is believed that any breakthrough could greatly promote the commercialization and industrialization of this promising technique.

## Figures and Tables

**Table 1:** Several representative types of reactive electrochemical membrane (REMs).

Type of REMs	Pore Size	Blank Control	Mass Transfer Enhancement	Electro-Active Area Increasement	Reference
A seepage carbon nanotube electrode		Without insulated porous membrane	1.6-fold		[44]
Electrochemical carbon nanotube (CNT) filter	40–50 μm	Same electrode in batch mode	6-fold		[86]
Substoichiometric titanium dioxide (Ti_4_O_7_) REM	1–6 μm	Same electrode without permeate	10-fold	619-fold	[45]
MnO_2_/Ti REM	≈10 μm	Same electrode without permeate	≈4-fold ^1^		[87]
RuO_2_/Ti REM	0.98 μm	Same electrode in batch mode	2-fold	2-fold	[57]
A multi-walled carbon nanotube/graphite-RuO_2_ REM	22 µm	Same electrode without permeate	4.6-fold		[62]
MnO_x_/Ti REM		Same electrode without permeate	≈3.8-fold		[88]

^1^ means no exact value given, value was analyzed from the data; Slash means no data.

**Table 2:** Recent studies of application of electrochemical anodic membranes (EAM) (REM).

Type of REM	Treating Subject Properties	Operation Conditions	Performance	Current Efficiency (CE)/Energy Consumption (EC)	Reference
A seepage carbon nanotube electrode	Simulated dye wastewater containing 25–200 mg/L Reactive Brilliant Red X-3B	U = 5–15 V C_electrolyte_ = 0–3 gL^−^^1^ Na_2_SO_4_ pH = 2–10V = 80.2 mLmin^−^^1^	Total color and COD were removed by 94.4% and 57.6%, respectively in 90 min, much higher than that of 32.8–37.4% and 28.0–32.7% removal by conventional electrochemical processes	CE = 33.1% at time of 45 min while others were 7.5%, and 5.3% EC = 101.34 kWh kg^−^^1^COD	[44]
TiO_2_/Carbon REM	Oily water with concentration of 200 mg/L	U = 2.0 V I = 10.0 mA C_electrolyte_ = 15 g L^−^^1^ Na_2_SO_4_ Q = 100 Lm^−^^2^h^−^^1^bar^−^^1^	Oil and COD removal were up to 86.2% and 94.4%, higher than original carbon membrane and TiO_2_/carbon membrane	EC = 0.166 kWh per ton of water	[21]
	Simulated phenolic wastewater with concentration of 10 mM	C_electrolyte_ = 15 g L^−^^1^ Na_2_SO_4_ J = 0.3 mA cm^−^^2^ pH = 6 Rt = 0–5.2 min	A high phenol removal rate and complete mineralization fraction of 99.96 and 72.4% were achieved		[48]
MnO_2_/Ti REM	Producing propionic acid by oxidation of n-propanol (160 ± 5 mmol L^−^^1^)	U = 2.8 V C_electrolyte_ = 15 g L^−^^1^ Na_2_SO_4_ T = 25 and 50 °C Rt = 0–22.55 min	*n*-propanol conversion and the selectivity to propionic acid were improved to 60.77% and 56.82% when Rt increased from 0 min to 22.55 min, and meanwhile their value were 98.44% and 79.33% when T raising from 25 to 50 °C		[46]
Substoichiometric TiO_2_ REM	Simulated industrial wastewater containing 1 mM *p*-methoxyphenol (*p*-MP)	C_electrolyte_ = 10 mM Na_2_SO_4_ J = 0–1.0 mA cm^−^^2^ V = 600 mL min^−^^1^ T = 21 ± 2 °C	Best *p*-MP and COD removal rate were 99.9 ± 0.17% and 30.1 ± 3.1%	CE > 73.3 % and best CE was 99.0 % at 0.5 mA cm^−^^2^	[45]
Ultrafiltration TiO_2_ Magneéli Phase REM	Simulated wastewater containing 1 mM Oxalic Acid; Another Simulated wastewater containing 9 mM ClO_4_^−^ and 10 mM NO_3_^−^	For oxidation of OA: U = 2.94 V Q = 390 LMH T = 21 °C C_electrolyte_ = 10 mM Na_2_SO_4_ For separation of Oxyanions: U = 0–10 V Q*J* = 58 and 1291 LMH	The optimal removal rate for oxalic acid was 401.5 ± 18.1 mmol h^−^^1^m^−^^2^ at 793 LMH; The removal rate of oxyanion was 67% at 58 LMH	EC for separation of oxyanions was 0.22 kWh m^−3^	[49]
Multi-walled carbon nanotubes (MWCNTs)–Ti_4_O_7_ Composite REM	Synthetic solutions containing 10 μM or 150 μM N-nitrosodimethylamine (NDMA)	U = −1.1 V/SHE cathodic potential Q = 100 or 200 LMH C_electrolyte_ = 10 mM NaH_2_BO_3_ pH = 8.0 ± 0.1	For 10 μM NDMA, the removal rate was below the HPLC method detection limit (0.1 μM), and GC/MS gives a value of approximate 4-log removal (99%); For 10 mM NDMA, the removal rate was 82.5 ± 1%	EC values were 0.12 ± 0.03 kWh m^−^^3^ and 0.58 ± 0.02 kWh m^−3^, respectively for 10 and 150 μM NDMA	[53]
RuO_2_/Ti REM	Simulated wastewater containing 20–100 mg/L Tricyclazole	J = 0–20 mA cm^−^^2^ C_electrolyte_ = 5 g L^−^^1^ Na_2_SO_4_ pH = 7 T = 20 °C V = 8 mL s^−^^1^	The removal rate of Tricyclazole was approximate 100% at each C_initial_, all higher than conventional plate electrode	Best CE was 61.07% at 3 mA cm^−^^2^	[57]
	Actual anticancer drugs wastewater containing 61.2 mg L^−^^1^ 5-Fluoro-2-Methoxypyrimidine	V = 0.08–0.31 mLmin^−^^1^ pH = 2–9 C_electrolyte_ = 0–7.5 g L^−^^1^ Na_2_SO_4_ J = 3–5 mA cm^−^^2^	COD and 5-Fluoro-2-Methoxypyrimidine of the wastewater were removed by 84.1% and 100% at optimal condition, while BOD_5_/COD value and EC_50,48h_ value were increased from 0.14 and 16.4% to 0.53 and 51.2%, respectively	EC = 1.5 kWh kg^−^^1^ COD	[58]
	Actual triazole fungicides discharged water in pilot scale (capacity of 10 m^3^ d^−1^) containing 150–200 mg L^−1^ Tricyclazole, 50–75 mg L^−1^ 1H-1,2,4-Triazole and 25–55 mg L^−1^ Propiconazole	J = 1.5–5.5 mA cm^−^^2^ pH = 3–9 V = 3 m^3^ h^−^^1^	Tricyclazole, 1H-1,2,4-Triazole and Propiconazole were removed by 94.19%, 90.11% and 100%, the COD of discharged water was removed by 53.06%, while the BOD_5_/COD ratio raised from 0.028 to 0.46	Operation cost was 0.85 $ (m^−^^3^ d^−^^1^)	[14]
Boron-doped multi-walled carbon nanotubes REM	Simulated wastewater containing 1 mg L^−^^1^ bisphenol A	U = 0 and 3 V C_electrolyte_ = 10 mM Na_2_SO_4_ pH = 3–9 V = 2 mL min^−^^1^	Nearly complete removal of 1 mg L^−1^bisphenol A at 2 and 3 V of applied DC potentials was achieved	CE was ranged from 120 to 140 %, while EC was ranged from 15 to 50 KWh Kg^−^^1^ under different operation conditions.	[92]
Graphite–REM	Simulated drinking water containing 0.4–40 mM sulfadiazine (SDZ) and natural waters containing 40 mM SDZ	U = 0.5–3.0 V Q = 25, 50, and 75 LMH C_electrolyte_ = 50 mM Na_2_SO_4_ pH = 7	For simulated drinking water, SDZ can be removed by approximate 100% at 3V. However, degradation rate of flow mode slower than batch and circulation mode, but it degraded more SDZ on the base of mass balance calculations; For natural waters containing SDZ, 79% SDZ was removed	EC value ranging from 0.007 to 0.39kWh m^−^^3^ for different voltages (0.5–3.0 V), and 0.14 to 0.37 kWh m^−^^3^ for different fluxes (25–75 LWH)	[99]
Bi-doped SnO_2_−Ti_n_O_2n−1_ REM	Simulated wastewater containing 1mM Terephthalic acid (TA), 10 μM Atrazine (ATZ) and 10 μM Clothianidin (CDN)	U = 2.1–3.5 V/SHE V = 0.5 mL min^−^^1^ T = 21 ± 2 °C C_electrolyte_ = 10 mM KH_2_PO_4_ pH = 4.5	TA and COD conversion were achieved > 99.9% and > 97% at 3.5 V; ATZ conversion and %N mineralization were achieved > 99.9% and 91.3% at 3.5 V; CDN conversion and %N mineralization were achieved > 99.9 and 96.5% at 3.5 V	The minimal EC values per log removal of < 0.53 kWh m^−^^3^ for TA, < 0.42 kWh m^−^^3^ for ATZ, and 0.83 kWh m^−^^3^ for CDN	[52]
TiO_2_@SnO_2_−Sb/Ceramic REM	Simulated wastewater containing 10 μM *p*-chloroaniline(PCA)	U = 1–5 V T = 25 ± 1 °C pH = 7.0 C_electrolyte_ = 50 mM Na_2_SO_4_ Q = 11.6−138.9 Lm^−^^2^ h^−^^1^	PCA was removed by 97.9% at voltage of 5V with flux of 17.4 L m^−^^2^ h^−^^1^ in flow-through mode, 1.9 times than that of flow-by mode. In addition, either the removal rate or mass transfer rate constant (km) was higher in flow-through mode	EC value at 4.0 and 5.0 V reached 8.6 and 23.1 Wh L^−^^1^, respectively, 5.9 and 15.9 times that of 3.0 V (1.5 Wh L^−^^1^)	[69]
Pd-Based REM	Simulated wastewater containing 1.0 mM NO_3_^−^	V = 0.2 and 1.8 mL min^−^^1^ U = −2.5 V/SHE	Concentration of NO_3_^−^ was lower than EPAs regulatory MCL (700 μM) after a short time treating (≈2 s)	EC value of treated surface water was 1.1 to 1.3 kWh mol^−^^1^ for 1 mM NO_3_^−^	[51]
Ti_4_O_7_-based REM	Simulated wastewater containing 1.4 g L^−1^ algal cell	I = 100–500 mA t = 30–120 min	Algal cells exhibited significant disruption, while lipid extraction efficiency increased by 1.5 times for treated algae (*p* < 0.05)		[93]
Titanium sub-oxide REM	Simulated wastewater containing ~10^6^ CFU/mL *Escherichia coli* (*E. coli*) and ~10^11^ plaque forming units (PFU)/mL bacteriophage MS2	C_electrolyte_ = 0.05 M Na_2_SO_4_ J = 0–10 mA cm^−^^2^ V = 5 mL min^−1^	*E. coli* decreased from 6.46 log CFU/mL to 0.18 log CFU/mL, while bacteriophage MS2 achieved 6.74 log reduction as compared to original concentration (1011 PFU mL^−^^1^)		[94]
Activated carbon fiber cloth-REM	Simulated wastewater containing 10^7^ cellsmL^−1^ Escherichia coli (*E. coli*)	C_electrolyte_ = 50 mM Na_2_SO_4_ V = 1–20 mL min^−^^1^ U = 0–20 V	Disinfection was enhanced to 0.5, 1.4, 7.3, and 7.3 log reduction for the applied voltages of 2, 5, 10, and 20 V, respectively, and the log reduction of 7.3 represented complete disinfection	EC value was 1.5 kWh m^−^^3^ for a complete disinfection	[95]
Activated carbon fiber felt-REM	Simulated wastewater containing 10^6^–10^7^CFU mL^−1^ Escherichia coli ATCC 25922 (*E. coli*)	C_electrolyte_ = 10 mM Na_2_SO_4_ Q = 100 Lm^−^^2^ h^−^^1^ U = 2.5 V	≈100% log removal efficiency was obtained at a low voltage of 2.5 V. Meanwhile, the system can maintain long-lasting bacterial disinfection efficiency of real wastewater (≈100% log removal) in continuous flow tests with *J* of 100 Lm^−^^2^ h^−^^1^		[96]
Moving-bed electrochemical membrane bioreactor (Anode as REM)	Simulated wastewater containing 100 μgL^−1^ sulfamethoxazole (SMX)	U = 2 V cm^−^^1^	Removal of SMX achieved at 88.8 ± 2.4% during 91 d operation, while COD and NH_4_^+^-N removal were 93.7 ± 2.6% and > 95%		[66]
β-PbO_2_-tubular reactive filter(TRF)	Surface water and municipal sewage treatment plant (MSTP) final effluent containing 0.5 or 0.6 mM Norfloxacin (NOR) and sulfamethoxazole (SMZ)	V = 3.57 × 10^−^^3^ m s^−^^1^ I = 0.05, 0.1–0.25 A	90% NOR degradation were achieved with Rt of 2.0 and 3.2 s for reservoir water (0.05A) and MSTP effluent (0.25 A); Effective removal for SMZ achieved with Rt of 4.1–5.4 s	0.005–0.024 kWh m^−^^3^ for NOR and 0.012–0.017 kWh m^−^^3^ for SMZ	[97]
Ti/SnO_2_-Sb REM	Simulated wastewater containing 20 μg L^−^^1^ stavudine	C_electrolyte_ = 10 mM Na_2_SO_4_ J = 2–10 mA cm^−^^2^ pH = 3.0–11.0	Stavudine could be 100% removed by variety conditions (current density > 8 mA cm^−^^2^, pH < 5)	Ranging from 0.87 to 2.29 Wh L^−^^1^ for 90% stavudine degradation	[98]

Celectrolyte represents concentration of electrolyte, U represents voltage, V represents flow rate, I represents current, Q represents flux, J represents current density, T represents temperature, Rt represents retention time, t represents time.

**Table 3:** Recent studies of application of electrochemical cathodic membranes (ECMs).

Type of ECM	Treating Subject Properties	Wastewater Characteristics	Technology	Operation Conditions	Performance	Reference
Carbon–PTFE cathode	Leachate concentrates from a municipal landfill site	3896 mg L^−1^ COD; 1347 mg L^−1^ TOC; 23.4 mS cm^−1^ conductivity pH = 7.70	EF	Undivided reactor: 200 mL 0.4 L min^−1^ oxygen flow rate 1–40 mM FeSO_4_ J = 30 mA cm^−2^ pH = 2–5	The removal efficiencies of TOC and TN were 82% and 51% within 6 h	[122]
Carbon–PTFE cathode	Leachate concentrate collected from a municipal landfill site (Beijing, China)	6635 mg L^−1^ COD 1650 mg L^−1^ TOC 50.2 mS cm^−1^ conductivitypH = 8.07	E-peroxone	0.3 L min^−1^ O_2_ and O_3_ mixture airflow rate I = 350 mA	87% of TOC was removed after 4 h	[123]
Carbon–PTFE cathode	Surface water collected from a reservoir in the suburban area of Beijing	2.53 mg L^−1^ DOC 0.037 cm^−1^ UV_254_ 243 uS cm^−1^ conductivity 4.68 mg L^−1^ Cl^−^ pH = 8.03	E-peroxone	Undivided reactor: 600 mL 150 mL min^−1^ O_2_/O_3_ flow rate J = 1.25–5.0 mA cm^−2^	Accelerated micropollutant abatement in the surface water and all micropollutants were completely removed within 10 min	[124]
Carbon–PTFE air-diffusion cathode	Olive oil mill wastewater collected from a pre-Mium extra virgin olive oil production mill in northeastern Spain	581.1 ± 2.3 mg L^−1^ TOC 3.50 mS cm^−1^ conductivity pH = 6.83 ± 0.07	Sequential EC/PEF	Undivided reactor: 200 mL magnetic bar at 700 rpm; 0.50 mM Fe^2+^ J = 25 mA cm^−2^ pH = 3 1 L min^−1^ air pumped	97.1% TOC was removed after 600 min with 115.8 kWh (kg TOC)^−1^	[125]
Carbon–PTFE air-diffusion cathode	Real wastewater (RWW) obtained from the secondary decanter of a municipal WWTF near Barcelona	81.1 mg L^−1^ total carbon 10.8 mg L^−1^ TOC 0.20 mg L^−1^ Fe^2+^ 2.20 mS cm^−1^ conductivity pH = 8.10	Solar PEF	A 2.5L flow plant operating in batch mode 30–35 W m^−2^ UV irradiance	A complete removal of parabens in 180 min and 66% mineralization at 240 min. The mineralization current efficiency reported was up to 1000%, with a low energy consumption of 84 kWh (kg TOC)^−1^	[126]
Carbon–PTFE air-diffusion cathode	Urban wastewater was collected from the secondary effluent of a wastewater treatment facility located in Gavà-Viladecans (Barcelona, Spain)	15.0 mg L^−1^ TOC 318.1 mg L^−1^ Cl^−^ 0.19 mg L^−1^ Fe^2+^ 3.2 mS cm^−1^ conductivity pH = 7.90	PEF	An undivided, cylindrical, double-jacketed tank reactor of 150 mL 5 W m^−2^ UVA irradiance	96% TOC reduction achieved in 0.050 M Na_2_SO_4_ of IrO_2_ based DSA^®^	[127]
Carbon–PTFE air-diffusion cathode	The raw wastewater to be spiked with synthetic food azo dyes was a secondary effluent obtained from a WWTP located in Gavá-Viladecans (Barcelona, Spain)	15 mg L^−1^ DOC 66 mg L^−1^ TN 1.3 mM SO_4_^2−^ pH = 7.50	Solar PEF	An undivided, cylindrical two-electrode glass cell with volume of 130 mL 0.50 mM Fe^2+^ 5 W m^−2^ UVA light at 360 nm	PEF-BDD is able to yielding almost total mineralization in a real water matrix (95% DOC removal)	[128]
Carbon–PTFE air-diffusion cathode	The secondary effluent of a WWTP located in Gavà-Viladecans (Barcelona, Spain)	12.2 mg L^−1^ TOC 1.73 mS cm^−1^ conductivity 0.19 mg L^−1^ Fe^2+^ 318 mg L^−1^ Cl^−^ 141.3 mg L^−1^ SO_4_^2−^pH = 8.10	PEF	Undivided cell: 150 mL 1 L min^−1^ air flow rate 0.05 mM Fe^2+^ 5 W m^−2^ UVA light (λ_max_ = 360 nm)	Completely removal of tetracaine in 90 min. A 78% TOC abatement was found at 11 h, while 100% mineralization at 24 h	[129]
